# Epidemiology of Chikungunya Virus Outbreaks in Guadeloupe and Martinique, 2014: An Observational Study in Volunteer Blood Donors

**DOI:** 10.1371/journal.pntd.0005254

**Published:** 2017-01-12

**Authors:** Pierre Gallian, Isabelle Leparc-Goffart, Pascale Richard, Françoise Maire, Olivier Flusin, Rachid Djoudi, Jacques Chiaroni, Remi Charrel, Pierre Tiberghien, Xavier de Lamballerie

**Affiliations:** 1 Etablissement Français du Sang (EFS) Alpes Méditerranée, Marseille, France; 2 Aix Marseille Université, IRD U190, INSERM U1207, EHESP: "Emergence des Pathologies Virales", Marseille, France; 3 Institut Hospitalo Universitaire (IHU) Méditerranée Infection, Marseille, France; 4 Etablissement Français du Sang (EFS) La Plaine-Saint-Denis, France; 5 Centre National de Référence (CNR) des Arbovirus, Institut de Recherche Biomédicale des Armée, Hôpital d’Instruction des Armées Laveran, Marseille, France; 6 Etablissement Français du Sang (EFS) Martinique, Fort de France, France; 7 Etablissement Français du Sang (EFS) Guadeloupe-Guyane, Point a Pitre, France; 8 Université de Franche- Comté, Besançon, France; Oswaldo Cruz Foundation, BRAZIL

## Abstract

**Background:**

During Dec-2013, a chikungunya virus (CHIKV) outbreak was first detected in the French-West Indies. Subsequently, the virus dispersed to other Caribbean islands, continental America and many islands in the Pacific Ocean. Previous estimates of the attack rate were based on declaration of clinically suspected cases.

**Methods/Principal findings:**

Individual testing for CHIKV RNA of all (n = 16,386) blood donations between Feb-24^th^ 2014 and Jan-31^st^ 2015 identified 0·36% and 0·42% of positives in Guadeloupe and Martinique, respectively. The incidence curves faithfully correlated with those of suspected clinical cases in the general population of Guadeloupe (abrupt epidemic peak), but not in Martinique (flatter epidemic growth). No significant relationship was identified between CHIKV RNA detection and age-classes or blood groups. Prospective (Feb-2014 to Jan-2015; n = 9,506) and retrospective (Aug-2013 to Feb-2014; n = 6,559) seroepidemiological surveys in blood donors identified a final seroprevalence of 48·1% in Guadeloupe and 41·9% in Martinique. Retrospective survey also suggested the absence or limited "silent" CHIKV circulation before the outbreak. Parameters associated with increased seroprevalence were: Gender (M>F), KEL-1, [RH+1/KEL-1], [A/RH+1] and [A/RH+1/KEL-1] blood groups in Martiniquan donors. A simulation model based on observed incidence and actual seroprevalence values predicted 2·5 and 2·3 days of asymptomatic viraemia in Martiniquan and Guadeloupian blood donors respectively.

**Conclusions/Significance:**

This study, implemented promptly with relatively limited logistical requirements during CHIKV emergence in the Caribbean, provided unique information regarding retrospective and prospective epidemiology, infection risk factors and natural history of the disease. In the stressful context of emerging infectious disease outbreaks, blood donor-based studies can serve as robust and cost-effective first-line tools for public health surveys.

## Author Summary

Chikungunya virus (CHIKV) is an emerging mosquito-borne arbovirus responsible of a large outbreak since December 2013 in the Americas from French islands in the Caribbean. Documentation of the epidemic was based on the survey of clinically suspected cases, providing limited information on the incidence of the disease overtime and the herd immunity of the general population at the end of the outbreak. Our study improved blood donors specimen collection and data obtained from the Nucleic Acid Testing (NAT) screening implemented during the outbreak in order to prevent CHIKV transmission by blood products. After an 11 month follow up, we determine for Martinique and Guadeloupe islands the CHIKV-RNA positive rate: 0.42% and 0.36% respectively and the final IgG seroprevalence: 41.2% and 48.1%. Using a simulation model, we estimate the CHIKV duration of asymptomatic viremia to be between 2.3 and 2.5 days. Our findings will help in the comprehension of the natural history of infection and provide helpful data for prevention of Transfusion transmitted infections. Our study provides evidence that monitoring of Chikungunya infection based on NAT screening of voluntary blood donors can be implemented rapidly and provides real-time epidemiological information. This should be of specific relevance to the case of epidemics caused by viral infections with high numbers of asymptomatic forms such as observed with the currently emerging Zika virus.

## Introduction

Chikungunya virus (CHIKV), an *Aedes*-borne alphavirus first identified in Tanzania in the early 1950's, infects humans through a "zoonotic cycle" (*i*.*e*., starting from a non-human primate reservoir and sylvatic mosquitoes) or a "dengue-like cycle" (*i*.*e*., via direct human-mosquito-human transmission by peridomestic *Aedes aegypti* and *Ae*. *albopictus* mosquitoes). During the past decade the epidemic transmission cycle of CHIKV has caused large outbreaks throughout Asia, Africa and the islands in the Indian Ocean. The disease is usually mild and characterised by acute febrile arthralgia. Severe forms of infection have been reported, notably encephalitic syndromes in newborns following late infection of the mother during pregnancy. In addition, debilitating persistent arthralgic sequelae are observed in a proportion of patients [1].

In December 2013, the first autochthonous cases of chikungunya fever in the Americas were recorded in the French-Dutch Caribbean Saint-Martin Island [2]. Subsequently, the virus spread to other islands of the French West Indies (Saint-Barthelemy, Martinique and Guadeloupe), to the majority of Caribbean islands and to continental America. By now, this episode has probably involved more than one million people [3].

In the most populated French Caribbean islands (Guadeloupe and Martinique), the only potential vector of CHIKV is *Ae*. *aegypti*. This species is abundant and also responsible for dengue virus epidemics [4]. It was therefore anticipated that *Ae*. *aegypti* would transmit CHIKV locally. Indeed, in 2014, at least 81,200 presumed clinical cases of chikungunya fever were recorded in Guadeloupe, and 72,500 in Martinique [5]. Consequently, special attention was paid to minimizing the risk of virus transmission via blood transfusion. However, a temporary ban on local blood donation would have presented a major challenge for supplies of fresh blood products from France, due to local phenotypic distribution of blood groups. Accordingly, CHIKV-specific molecular screening was implemented by the French blood bank (Etablissement Français du Sang, EFS) [6] and collections of human sera were provided by EFS for serological analyses.

Here, we report an epidemiological follow-up of the chikungunya outbreak in Guadeloupe and Martinique islands, based on the large-scale prospective molecular detection of incident cases in blood donors and on seroprevalence analyses performed in donors at different time intervals during the epidemic.

## Materials & Methods

### Ethics statement

Only volunteer blood donors were included. All of them were specifically informed that samples would be tested for blood-borne pathogens in order to prevent transfusion-transmitted infections and also might be used for epidemiological studies. They provided signed written informed consent. The study was approved by the scientific direction of the EFS. No specific sampling dedicated to the study was performed. All data used for epidemiological studies were de-identified.

### Demographic data

In 2014, 403,750 inhabitants were living in Guadeloupe (sex ratio = 0·86) and 381,326 in Martinique (sex ratio = 0·85) [7]. The distribution according to age-groups and gender is available in S1 Fig (supporting information section).

### Study design and population studied

#### (i) Population

In Guadeloupe and Martinique EFS conducts blood collection, processing and distribution from non-remunerated volunteers blood donors (18-70yo) recruited according to French regulatory requirements. In 2014, the medical pre-donation questionnaire included a description of common CHIKV clinical symptoms. Blood donors with diagnosis or suspicion of chikungunya fever were deferred for 28 days following recovery.

Donors recruited in the study were assigned to one of five age-groups (18–30, 31–40, 41–50, 51–60, and 61–70). The information made available for analysis included date of donation, sex, age, birth place and results for ABO, Rhesus and Kell blood phenotyping. For each donor, the number of blood donations over the period was recorded.

#### (ii) Molecular study

**Fig 1:**
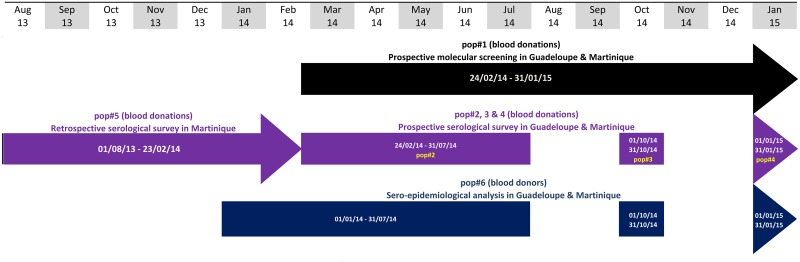
Flow chart of sample collection.

Between Feb-24^th^ 2014 and Jan-31^st^ 2015, all blood donations in Guadeloupe (n = 6,189; sex ratio = 0·96) and Martinique (n = 10,197; sex ratio = 0·88) were subjected to individual nucleic acid testing (NAT) for CHIKV RNA (n = 16,386; sex ratio = 0·91; pop#1; Fig 1).

#### (iii) Seroprevalence analyses

Our objective was to follow the rise of seroprevalence at the onset of the outbreak, to estimate the final seroprevalence, and to refine the picture of epidemic kinetics and help calibrating our model by obtaining an intermediate seroprevalence value. Accordingly, a sero-epidemiological survey was conducted using a selection of samples (n = 9,506) collected for NAT screening. This included donations collected in Guadeloupe and Martinique between Feb-24^th^ and July-31^st^ 2014 (n = 6,812; pop#2; Fig 1), randomised samples in October 2014 (n = 940; pop#3; Fig 1) and January 2015 (n = 1,754; pop#4; Fig 1). Seroprevalence was analysed separately for Guadeloupe and Martinique.

In addition, a retrospective sero-épidemiological study was performed from donations collected in Martinique island between Aug-1^st^ 2013 and Feb-23^th^ 2014 (n = 6,559; pop#5; Fig 1) in order to identify possible CHIKV circulation before the first description of autochthonous cases.

A summary of the characteristics of the different groups studied is available in S1 Table (supporting information section).

### Biological testing

#### (i) Blood grouping

ABO/Rh/Kell phenotypes were determined using a fully automated microplate haemagglutination procedure (Immucor Galileo System) according to routine EFS procedures.

#### (ii) NAT screening

Individual NAT screening was performed by the EFS Alpes-Méditerranée laboratory (Marseille, France). Nucleic acids were extracted from 140μL of plasma using the NucleoSpin-96 Virus Extraction Kit (Macherey-Nagel) and a semi-automated NX-workstation (Beckman-Coulter). Real-time RT-PCR amplification was realised with the RealStar Chikungunya RT-PCR Kit 1.1 (Altona Diagnostics) and a CFX96 thermocycler (Bio-Rad) [6].

The performance of the assay was evaluated by testing serial dilutions of quantified synthetic control RNAs provided by the manufacturer. In addition, serial dilutions of supernatants of Vero cells infected by the Asian and ECSA lineages of CHIKV were tested in quadruplicate using the same extraction kit and the same amplification protocol as those used for NAT screening of blood donors (biological material provided by the National Reference Center for Arboviruses).

#### (iii) Serological testing

ELISA detection of CHIKV-specific IgG was performed at the National Reference Centre for Arboviruses (French Army, Marseille, France) as previously described [8]. Serology tests were performed from individual plasma samples, except for population#5 (retrospective analysis corresponding to the early phase of the outbreak in Martinique) which was performed using minipools containing 5 donations (and subsequent individual testing when a positive pool was detected).

### Statistical analyses

Association of the presence of CHIKV-IgG with other epidemiological and biological factors was analysed using records from 8,653 donors tested during the epidemic period (Jan-1^st^ 2014 to Jan-31^st^ 2015), in both Guadeloupe (2,984, sex ratio = 0·94) and Martinique (5,669, sex ratio = 0·81) (pop#6; Fig 1, see S1 Table for details), and tested using the Chi-square test. To avoid possible bias, when a donor was associated with several blood donations during the period considered, he/she was counted only once (the day of the first donation if serology remained negative, otherwise the day of the first positive serology). Analyses were performed separately for Guadeloupe and Martinique. The main parameters considered were gender, age and blood grouping phenotypes. Statistical analysis relied on Chi^2^ analysis were performed online using "Chi-Square Test Calculator" (http://www.socscistatistics.com/tests/chisquare2/Default2.aspx). Multivariate analysis was performed using binary logistic regression with the IBM-SPSS Statistics v 23.0.0.0 software. Results were considered statistically significant when p-value was lower than 0·05.

### Simulation modelling

The population of blood donors tested by NAT screening for CHIKV (blood donations = pop#1) in Martinique included 6,911 donors. The complete duration of the study in pop#1 was 338 days, including 224 days open for blood donation. Accordingly, during these 224 days, our model randomly attributed 1 or 2 days of possible donation to 4,147 donors who gave blood once and 2,764 who gave blood twice, respectively. It also randomly attributed 1, 2, 3 or 4 days of viraemia to each donor in a 338 day period and the number of days where viraemia and blood donation coincided were counted. The mean value obtained in 1,000 simulation replicates provided the expected number of detected incident cases in Martiniquan pop#1 donors, assuming a 1–4 day duration of detectable asymptomatic viraemia and a final seroprevalence of 100%. Results were then adjusted in proportion with the actual seroprevalence observed at the end of the study period. The same analysis was performed in pop#1 Guadeloupian blood donors (4,613 donors, including 2,768 who gave once and 1,845 who gave twice). This model was used to determine which estimated duration of detectable asymptomatic viraemia provided the best fit to positive viral RNA detection following NAT screening. This estimate was therefore dependent upon the specific LOD of the detection method used.

## Results

### Blood grouping

In the population of donors used for sero-epidemiological analyses (#pop6), the distribution of ABO blood groups was: O: 54·7%; A: 27·4%; B: 14·9%, AB: 2·90%. The prevalence values of Rhesus positive (RH+1) and Kell positive (KEL+1) phenotypes were 88·2% and 4·1%, respectively.

### NAT screening of blood donors

The manual of the kit used in the current study indicated that the limit of detection (LOD) of the assay (Probit analysis based on serial dilutions of quantified synthetic control RNAs) was 1.268 copies/μL of eluate [95% confidence interval (CI): 0.610 copies/μl—4.053 copies/μl]. In our experimental conditions, this would correspond to a LOD of *ca* 450 genome copies per mL of plasma. Using the same control RNAs, our results were in a similar range (300–400 synthetic RNA copies per mL of plasma). Further evaluation using titrated culture supernatants allowed for both the Asian and ECSA lineages of CHIKV the reproducible detection of viral RNA in dilutions corresponding to a titre ≥ 1 TCID/mL.

**Fig 2:**
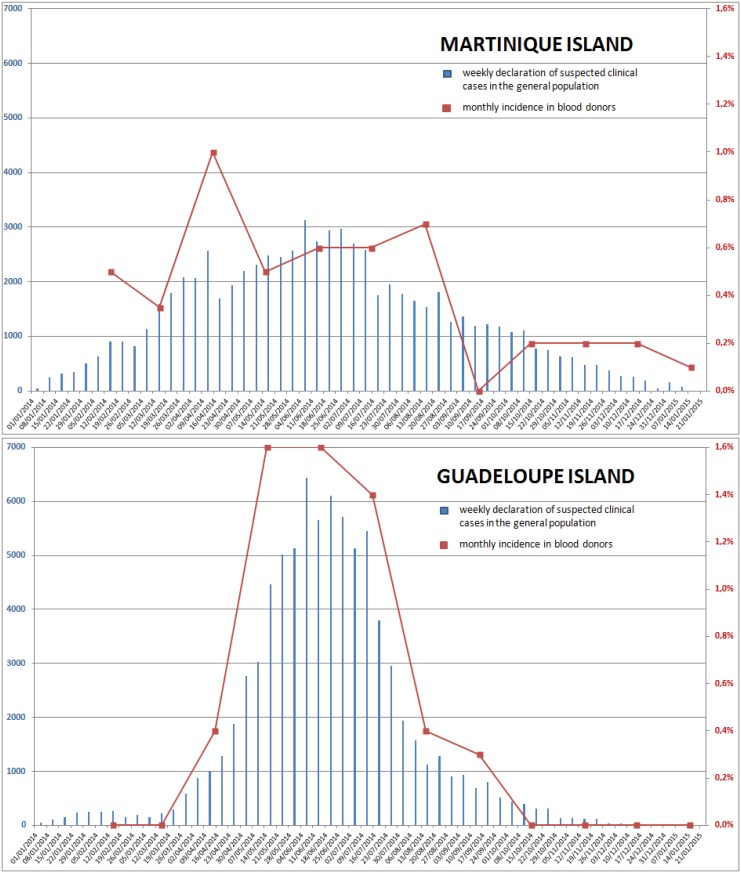
Temporal distribution of CHIK incident cases.

Amongst 16,386 donations tested by RT-PCR (pop#1), 37/10,197 (0·36%) and 26/6,189 (0·42%) were positive in Martinique and Guadeloupe respectively. Monthly detection of CHIKV RNA is presented in Fig 2, together with suspected clinical cases in the general population. None of the donors with a positive CHIKV RNA NAT screening result did repeat blood donation over the study period. The results clearly showed different epidemic kinetics in Guadeloupe and Martinique. The outbreak started earlier in Martinique (threshold of 1,000 weekly suspected clinical cases from early Mar-2014) than in Guadeloupe (same threshold from early Apr-2014) with a clear and very intense epidemic peak in Guadeloupe during May-Aug-2014 (up to 6,500 weekly cases, *ca*. 1,500/100,000 inhabitants) and a flatter curve in Martinique (peak at *ca*. 3,000 weekly cases, *ca*. 800/100,000 inhabitants).

The curve of incidence produced from CHIKV RNA detection in blood donations faithfully correlated with that of suspected clinical cases in the general population in Guadeloupe, but less accurately in Martinique. No significant statistical relationship between CHIKV RNA detection and age-classes or blood groups was identified.

### Seroprevalence analyses

**Fig 3:**
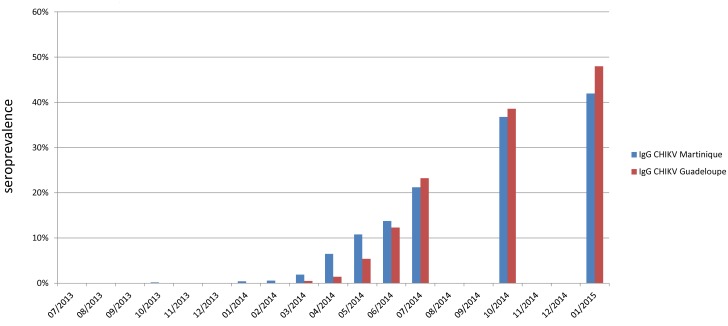
Evolution of CHIKV seroprevalence.

Monthly prevalence values of CHIKV-IgG in populations #2–5 are presented in Fig 3 for Guadeloupe and Martinique. This covers the period from Aug-1^st^ 2013 to Jan-31^st^ 2015 and includes both results from a prospective study (starting at the end of Feb-2014) and a retrospective study (Aug-2013 to Feb-2014) limited to Martinique.

**Fig 4:**
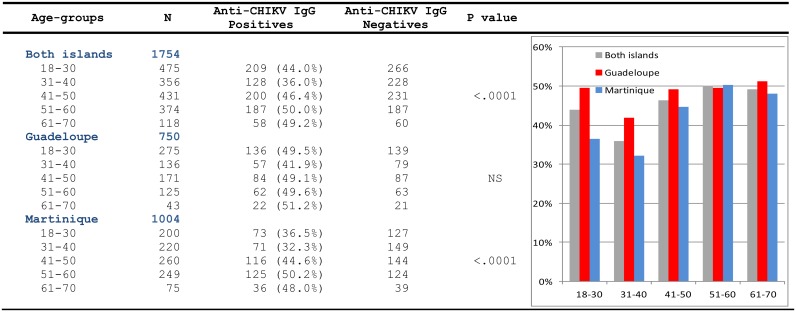
Distribution of CHIKV seroprevalence in age-groups at the end of the outbreak. Seroprevalence was measured in 1,754 donors sampled in January 2015 (pop#4). P values indicates that distribution of seroprevalence in age-groups is different from null hypothesis in the global population tested and in Martinique, but not in Guadeloupe where all values are close to 50%.

- The former identified a continuous prevalence increase in both islands with a final seroprevalence of 48·1% in Guadeloupe and 41·9% in Martinique (Jan-2015, pop#4). Fig 4 presents the distribution of positives in age-classes in pop#4, showing *(i)* in both islands the lowest values in the 31-40yo group, *(ii)* a trend to increase in the older age-groups and *(iii)* globally higher numbers in Guadeloupe than in Martinique.
  In the overall age range (18–70 yo) and after correction for the distribution of population in age-groups, seroprevalence estimates in the general population were calculated at 48·8% in Guadeloupe and 43·2% in Martinique. This presumably represents a valid proxy estimate for the global seroprevalence in the general population of both islands.
- The latter identified 2 Martiniquan donors with CHIKV-IgG collected in Oct-2013. One reported previous chikungunya fever during the 2005–2006 Indian Ocean outbreak; the other case could not be investigated. It could not be determined whether this was an autochthonous case, an imported case, or the serological signature of a previous local infection.

### Statistical analysis

Statistical analyses were performed using results collected from population #6, *i*.*e*. donors sampled during the outbreak (see Fig 1, Tables 1 and 2).

**Table 1:** Statistical analysis of risk factors associated with antibodies to CHIKV (univariate analysis).

Variables	N	Anti-CHIKV IgG Positives	Anti-CHIKV IgG Negatives	Test	P value
**Gender**					
** Both islands**	**8653**				
Male	3994	771 (19.3%)	3223	male > female	0.0015
Female	4659	777 (16.7%)	3882		
** Guadeloupe**	**2984**				
Male	1448	304 (21.0%)	1144	male vs female	NS
Female	1536	332 (21.6%)	1204		
** Martinique**	**5669**				
Male	2546	467 (18.3%)	2079	male > female	<0.0001
Female	3123	445 (14.2%)	2678		
**ABO blood group**					
** Both islands**	**8629**				
O	4726	872 (18.4%)	3854	O vs ≠O	NS
A	2366	397 (16.8%)	1969	A vs ≠A	NS
B	1286	233 (18.1%)	1053	B vs ≠B	NS
AB	251	43 (17.1%)	208	AB vs ≠AB	NS
**Guadeloupe**	**2976**				
O	1655	350 (21.1%)	1305	O vs ≠O	NS
A	826	175 (21.2%)	651	A vs ≠A	NS
B	433	88 (20.3%)	345	B vs ≠B	NS
AB	62	21 (33.9%)	41	AB vs ≠AB	0.0146
**Martinique**	**5653**				
O	3071	522 (17.0%)	2549	O vs ≠O	NS
A	1540	222 (14.4%)	1318	A vs ≠A	NS
B	853	145 (17.0%)	708	B vs ≠B	NS
AB	189	22 (11.6%)	167	AB vs ≠AB	NS
**Rhesus blood group**					
** Both islands**	**8629**				
RH+1	7611	1377 (18.1%)	6234	RH+1 vs -1	NS
RH-1	1018	168 (16.5%)	850		
** Guadeloupe**	**2976**				
RH+1	2635	565 (21.4%)	2070	RH+1 vs -1	NS
RH-1	341	69 (20.2%)	272		
** Martinique**	**5653**				
RH+1	4976	812 (16.3%)	4164	RH+1 vs -1	NS
RH-1	677	99 (14.6%)	578		
**Kell blood group**					
** Both islands**	**8629**				
KEL+1	358	52 (14.5%)	306	KEL+1 vs -1	NS
KEL-1	8271	1492 (18.0%)	6779		
**Guadeloupe**	**2976**				
KEL+1	117	26 (22.2%)	91	KEL+1 vs -1	NS
KEL-1	2859	607 (21.2%)	2252		
**Martinique**	**5653**				
KEL+1	241	26 (10.8%)	215	KEL+1 < -1	0.0270
KEL-1	5412	885 (16.3%)	4527		
**COMBINATIONS**					
**[RH+1 & KEL-1] blood groups**					
** Both islands**	**8629**				
[RH+1 & KEL-1]	7293	1335 (18.3%)	5958	[RH+1 & KEL-1]	0.0196
others	1336	209 (15.6%)	1127	> others	
**Guadeloupe**	**2976**				
[RH+1 & KEL-1]	2533	544 (21.5%)	1989	[RH+1 & KEL-1]	NS
Others	443	89 (20.1%)	354	vs others	
**Martinique**	**5653**				
[RH+1 & KEL-1]	4760	791 (16.6%)	3969	[RH+1 & KEL-1]	0.0177
Others	893	120 (13.5%)	773	> others	
A & Rhesus blood groups*					
**Both islands**	**2366**				
[A & RH+1]	2088	361 (17.3%)	1727	[[A & RH+1]	0.0482
[A & RH-1]	278	35 (12.6%)	243	vs [A & RH-1]	
**Guadeloupe**	**826**				
[A & RH+1]	730	154 (21.1%)	576	[A & RH+1]	NS
[A & RH-1]	96	20 (20.8%)	76	vs [A & RH-1]	
**Martinique**	**1540**				
[A & RH+1]	1358	207 (15.2%)	1151	[A & RH+1]	0.0158
[A & RH-1]	182	15 (08.2%)	167	> [A & RH-1]	
[A & RH+1 & KEL-1] blood group*					
**Both islands**	**2366**				
[A & RH+1 & KEL-1]	1983	347 (17.5%)	1636	[A & RH+1 & KEL-1]	0.0237
Other A	383	49 (12.3%)	334	> other A	
**Guadeloupe**	**826**				
[A & RH+1 & KEL-1]	698	147 (21.1%)	551	[A & RH+1 & KEL-1]	NS
Other A	128	27 (21.0%)	101	vs other A	
**Martinique**	**1540**				
[A & RH+1 & KEL-1]	1285	200 (15.6%)	1085	[A & RH+1 & KEL-1]	0.0054
Other A	255	22 (08.6%)	233	> other A	

* same tests NS for other ABO groups

**Table 2:** Statistical analysis of risk factors associated with antibodies to CHIKV (multivariate analysis). The table includes only risk factors associated with P value <0.05.

Variables	Test	P value	Odd ratio	95% confidence interval
**Gender** (both islands)	male gender increases risk	0.002	1.189	[1.065–1.328]
**Age** (both islands)	younger age protective	0.004	0.994	[0.990–0.998]

**(i) Univariate analyses** (Table 1):

- Detection of CHIKV-IgG was significantly more frequent in males than females in Martinique (p <0·0001) but not in Guadeloupe.
- No direct significant relationship was identified between ABO groups and detection of CHIKV-IgG, with the exception of higher CHIKV-IgG rates in Guadeloupian donors with an AB phenotype. This observation should be considered with caution since it was obtained from low numbers and only in Guadeloupe.
- Significantly higher CHIKV-IgG rates were observed in Martiniquan donors with a KEL-1 phenotype. Detection of CHIKV-IgG was more frequent in both islands in donors with an RH+1 phenotype, but this was not statistically significant.
- When combination phenotypes were tested, CHIKV-IgG detection was significantly more frequent in Martiniquan (but not in Guadeloupian) donors with:
  1. a [RH+1 & KEL-1] phenotype
  2. a [A & RH+1] phenotype
  3. a [A & RH+1 & KEL-1] phenotype

Equivalent associations with AB, O, and B groups were insignificant.

**(ii) Multivariate analyses** (Table 2):

The goodness of fit test of Hosmer and Lemeshow was 0·502. Significant association were identified at week level as follows (global analysis of both islands):

- Male gender was associated with an increased risk of seropositivity
- Seropositivity globally increased with age

### Duration of asymptomatic viraemia

For a final seroprevalence of 41·9%, in the pop#1 of Martiniquan blood donors our model predicted, 15, 31, 46, and 61 RNA positive detections (with a viral load above 450 genome copies/mL of plasma) associated with asymptomatic viraemia durations of 1, 2, 3, and 4 days respectively. Based on this model, the observed number of RNA positive detections (37) corresponded to a predicted asymptomatic viraemia of *ca*. 2·5 days.

For a final seroprevalence of 48·1% in the pop#1 of Guadeloupian donors, 12, 24, 35, and 47 RNA positive detections (with a viral load above 450 genome copies/mL of plasma) were associated with asymptomatic viraemia durations of 1, 2, 3, and 4 days respectively. Thus, the observed number of RNA positive detections (26) corresponded to a predicted asymptomatic viraemia of *ca*. 2·3 days.

## Discussion

It has been previously speculated that historical reports would suggest previous circulation episodes of Chikungunya virus in the Americas [3]. The only documented introduction of the virus (Asian genotype) in the region occurred at the end of 2013 [2] and has been responsible for large outbreaks in the Caribbean islands, and numerous countries of Central and South-America. Moreover, 12 Floridian autochthonous cases [9] were reported in 2014.

After the discovery of CHIKV clusters in Saint-Martin and Saint-Barthelemy islands (Dec-2013), the virus, transmitted by *Aedes aegypti* mosquitoes, dispersed rapidly to Martinique and later to Guadeloupe. In La Martinique, according to French health authorities the disease reached epidemic proportions on Jan-3^rd^ 2014, and the alert was cancelled on Jan-8^th^ 2015. On Guadeloupe the epidemic alert started on Apr-10^th^ 2014 and ended on Nov-27^th^ 2014. The epidemic kinetics were different on the two islands, with a shorter and more intense outbreak in Guadeloupe. The peak of clinically suspected cases occurred during week-23 in both Guadeloupe (*ca*. 6,500 cases) and Martinique (*ca*. 3,250 cases) [5]. The precise origin of the observed differences is unknown. They may be related to ecological and environmental factors (e.g., climatic and geographical conditions, density and distribution of mosquito and human populations, land use…), but also to anthropogenic factors such as pressure of vector control.

The current study was based on volunteer blood donors. Limitations to the interpretation of epidemiological data are therefore those of classical blood donor studies (individuals studied were 18–70 years old, and had no history of virus-like illness in the 28 days before donation). Important assets of the current study design were, the opportunity to compare results from similar populations in different locations, the high number of individuals enrolled, access to pre-epidemic samples, the possibility of performing individual nucleic acid tests and having access to asymptomatic or pre-symptomatic viraemiac individuals.

The first important finding relates to the occurrence of cases in Martinique before the detection of cases by the public health services (Dec-18^th^ 2013). Because the clinical symptoms of dengue and chikungunya fever are similar (flu-like disease), the ongoing dengue outbreak may have hidden the emergence of CHIKV and delayed the detection of the first cases. Our retrospective seroprevalence analysis identified only two donors with antibodies to CHIKV between August and December 2013 (both being detected in Oct-2013): one associated with previous infection during the Indian Ocean outbreak and the other that could not be investigated. Overall, this suggests the absence of significant circulation of the virus during several decades before the outbreak, and also the rapid detection of the first outbreak cases.

Another interesting observation was the impact of different epidemic kinetics on our ability to identify risk factors associated with antibodies to CHIKV. In Guadeloupe, the epidemic eruption was intense but the very high transmission rate was not associated with identifiable risk factors. By contrast, the flatter epidemic curve in Martinique correlated with an overrepresentation of males and individuals with [RH+1 & KEL-1], [A & RH+1] and [A & RH+1 & KEL-1] phenotypes and CHIKV-IgG (using univariate analysis). Being a female or having a blood group different from these risk factors was apparently "protective". However, this protection was relatively minor taking into account the overall situation in Guadeloupe. In multivariate analysis, the only significant associations identified were gender (risk increased in males *vs* females) and age (global increase of risk with age), both with low odd ratio values.

The observed higher risk of CHIKV infection in males has been previously reported [10–13]. However, in other studies higher prevalence in females has been reported [14–17]. The reasons underlying these divergent observations remain elusive and are presumably linked with different habitats, economic factors and lifestyles. Regarding blood types and association with CHIKV infection, there is very limited information available. Kumar *et al*. claimed that Rhesus-positive individuals had increased susceptibility to acquiring CHIKV infection [18], but their results did not support this. In a genetic predisposition study in 100 Indian families, Lokireddy *et al*. identified infection in all Rhesus-positive blood groups [19] but none amongst Rhesus-negative individuals. However, the proportion of Rhesus-negative individuals was too low to draw robust conclusions. The association between erythrocyte phenotypes and susceptibility to viral infection remains difficult to interpret as long as the mechanisms and cellular receptors involved in CHIKV infection are not fully identified. The risk associated with specific blood groups may be explained by several non-mutually exclusive factors: individuals with these blood groups may be more prone to mosquito bite (for biological, epidemiological or sociological reasons); alternatively, they may have different susceptibilities to infection or capacities to eliminate the virus via specific innate immune responses.

It is interesting to observe that on both islands the outbreak declined when a 40–50% herd immunity level was reached. This threshold is strikingly similar to that observed at the end of the outbreak on Reunion island (38·2%) and Mayotte (37·2%) [12,20]. This is despite the fact that different virus genotypes were implicated in the Caribbean and Indian Ocean epidemics. In contrast the threshold for decline was much higher in Kenya (72%), Kerala (68%) and Thailand (62·1%) [10, 13, 21]. This possibly reflects differing levels of social and economic development together with local efficacy of mosquito control measures. Based on the situation observed in the Indian Ocean countries, the level of immunity detected in the Martiniquan and Guadeloupian population should reduce the likelihood of a major recurrence of chikungunya fever in the French West Indies during the next few years.

Finally, concerning the implications for blood transfusion, our study clearly shows that the risk of collecting blood from asymptomatic viraemiac patients is significant during a chikungunya fever outbreak. Modelling performed in Martiniquan and Guadeloupian blood donors suggested that the duration of asymptomatic viraemia was close to 2.5 days. This estimated period may be extended by the use of molecular assays with an improved limit of detection. However our results are consistent with the post-donation survey of 48 viraemiac blood donors, in which clinical symptoms were reported 1–5 days after donation (39·6% at day 1, 39·6% at day 2, 14·5% at day 3, 4·2% at day 4 and 2·1% at day 5; mean value = 1·9 days). The actual mean duration of asymptomatic viraemia corresponds to this observed mean delay before the symptoms, plus the duration of viraemia prior to donation, *i*.*e*. it should be very close to the value provided by our model. This new information suggests that the optimal quarantine period for blood products during a chikungunya fever outbreak should be at least 5 days. The length of the asymptomatic viraemic period identified in the current study is higher than previous proposals (1·5 days according to Brouard *et al*.) [22]. This divergence could reflect either different durations of asymptomatic viraemia in the case of infection by CHIKV ECSA when compared with the Asian genotype, or underestimation of the actual duration due to the limited number of cases previously analysed.

In conclusion, this study demonstrates the ability of blood donor-based investigations to be implemented promptly even during an intensely rapid onset of virus emergence. It also provides both retrospective and prospective data relating to epidemiological characteristics and infection risk factors without the requirement for a *de novo* cohort, hospitalisation of patients and specific blood sampling. Moreover, by combining the biological and post-donation follow-up data we have gained new knowledge relating to the natural history of the disease.

In the stressful context of emerging infectious disease outbreaks, appropriate blood donor-based studies have now been shown to be excellent first-line tools for public health surveys. This is particularly applicable to situations in which the proportion of asymptomatic individuals is high and seroprevalence information is required to estimate the attack rate as, indeed, exemplified by the currently emerging Zika virus.

## Supporting Information

S1 Fig Demographic characteristics of Martiniquan and Guadeloupian general populations. (TIF)

S1 Table Populations studied. (DOCX)

S1 Checklist STROBE checklist. (DOCX)
