# Supplementary figures and images for: Epidemiology of Chikungunya Virus Outbreaks in Guadeloupe and Martinique, 2014: An Observational Study in Volunteer Blood Donors
Source: PLoS Negl Trop Dis. 2017 Jan 12;11(1):e0005254. doi: 10.1371/journal.pntd.0005254 (PMC5230756; doi:10.1371/journal.pntd.0005254)

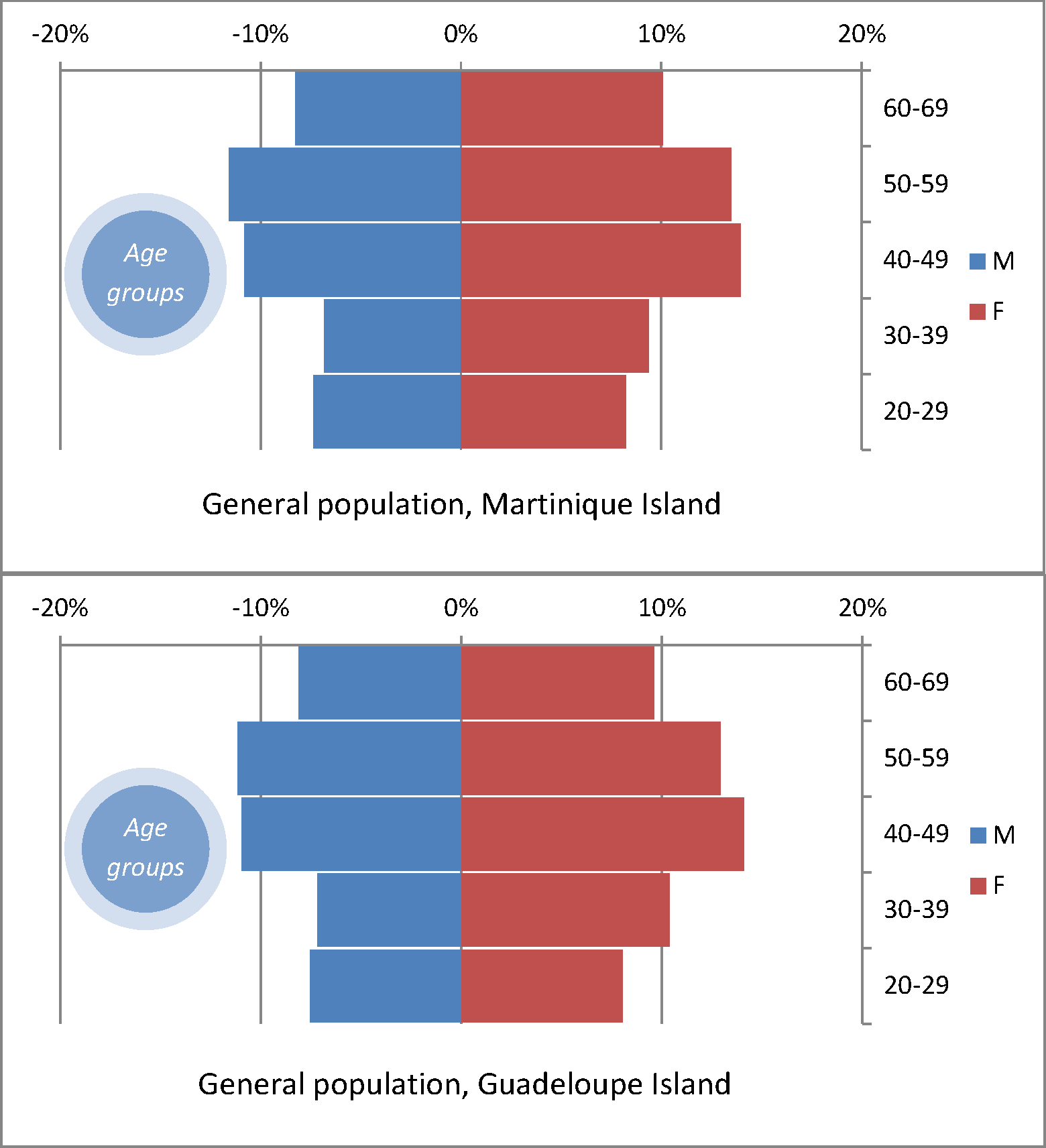

Supplement: S1 Fig — (TIF) [file pntd.0005254.s001.tif]
