# Supplementary material for: Epidemiology of Chikungunya Virus Outbreaks in Guadeloupe and Martinique, 2014: An Observational Study in Volunteer Blood Donors
Source: PLoS Negl Trop Dis. 2017 Jan 12;11(1):e0005254. doi: 10.1371/journal.pntd.0005254 (PMC5230756; doi:10.1371/journal.pntd.0005254)
Supplement: S1 Table — (DOCX) [file pntd.0005254.s002.docx]

ST1. Populations studied

|  |  |  |  |  |  |  |  |  |  |  |  |  |
| --- | --- | --- | --- | --- | --- | --- | --- | --- | --- | --- | --- | --- |
| Populations |  |  | All |  |  |  | Guadeloupe |  |  |  | Martinique |  |
|  |  | N | Male | Female |  | N | Male | Female |  | N | Male | Female |
|  |  |  |  |  |  |  |  |  |  |  |  |  |
| Pop#1 |  | 16,386 | 7,804 | 8,582 |  | 6,189 | 3,037 | 3,152 |  | 10,197 | 4,767 | 5,430 |
| Pop#2 |  | 6,812 | 3,290 | 3,522 |  | 2,435 | 1,205 | 1,230 |  | 4,377 | 2,085 | 2,292 |
| Pop#3 |  | 940 | 492 | 448 |  | 414 | 227 | 187 |  | 526 | 265 | 261 |
| Pop#4 |  | 1,754 | 814 | 940 |  | 750 | 351 | 399 |  | 1,004 | 463 | 541 |
| Pop#5 |  |  |  |  |  |  |  |  |  | 6,559 | 3,105 | 3,454 |
| Pop#6 |  | 8,653 | 3,994 | 4,659 |  | 2,984 | 1,448 | 1,536 |  | 5,669 | 2,546 | 3,123 |
|  |  |  |  |  |  |  |  |  |  |  |  |  |
|  |  |  |  |  |  |  |  |  |  |  |  |  |
