# Supplementary material for: Epidemiology of Chikungunya Virus Outbreaks in Guadeloupe and Martinique, 2014: An Observational Study in Volunteer Blood Donors
Source: PLoS Negl Trop Dis. 2017 Jan 12;11(1):e0005254. doi: 10.1371/journal.pntd.0005254 (PMC5230756; doi:10.1371/journal.pntd.0005254)
Supplement: S1 Checklist — (DOCX) [file pntd.0005254.s003.docx]

STROBE Statement—checklist of items **MANUSCRIPT(#PNTD-D-16-01047)**

**Epidemiology of Chikungunya Virus Outbreaks in Guadeloupe and Martinique, 2014: an Observational Study in Volunteer Blood Donors**

|  | | | Item No. | Recommendation | | | | Page  No. | | Relevant text from manuscript | |  |
| --- | --- | --- | --- | --- | --- | --- | --- | --- | --- | --- | --- | --- |
| **Title and abstract** | | | 1 | (*a*) Indicate the study’s design with a commonly used term in the title or the abstract | | | | 1 | | Observational Study in Volunteer Blood Donors | |  |
|  |  |  |  | (*b*) Provide in the abstract an informative and balanced summary of what was done and what was found | | | | 3-4 | | Individual testing for CHIKV RNA of all (n=16,386) blood donations between Feb-24^th^ 2014 and Jan-31^st^ 2015 identified 0·36% and 0·42% of positives in Guadeloupe and Martinique, respectively.  Prospective and retrospective seroepidemiological surveys in blood donors identified a final seroprevalence of 48·1% in Guadeloupe and 41·9% in Martinique.  A simulation model based on observed incidence and actual seroprevalence values predicted 2·5 and 2·3 days of asymptomatic viraemia in Martiniquan and Guadeloupian blood donors respectively. | |  |
| Introduction | | | | | | | | |  | | |  |
| Background/rationale | | | 2 | Explain the scientific background and rationale for the investigation being reported | | | | 5 | | In December 2013, the first autochthonous cases of chikungunya fever in the Americas were recorded in the French-Dutch Caribbean Saint-Martin Island. Subsequently, the virus spread to other islands of the French West Indies (Saint-Barthelemy, Martinique and Guadeloupe), to the majority of Caribbean islands and to continental America. By now, this episode has probably involved more than one million people. In the most populated French Caribbean islands (Guadeloupe and Martinique), the only potential vector of CHIKV is *Ae. aegypti*. This species is abundant and also responsible for dengue virus epidemics. It was therefore anticipated that *Ae. aegypti* would transmit CHIKV locally. Indeed, in 2014, at least 81,200 presumed clinical cases of chikungunya fever were recorded in Guadeloupe, and 72,500 in Martinique. Consequently, special attention was paid to minimizing the risk of virus transmission via blood transfusion. | |  |
| Objectives | | | 3 | State specific objectives, including any prespecified hypotheses | | | | 6 | | We report an epidemiological follow-up of the chikungunya outbreak in Guadeloupe and Martinique islands, based on the large-scale prospective molecular detection of incident cases in blood donors and on seroprevalence analyses performed in donors at different time intervals during the epidemic. | |  |
| Methods | | | | | | | | |  | | |  |
| Study design | | | 4 | Present key elements of study design early in the paper | | | | 7-8  Fig1 | | Non-remunerated volunteers blood donors (18-70yo) recruited according to French regulatory requirements.  Molecular study***.*** Between Feb-24^th^ 2014 and Jan-31^st^ 2015, all blood donations in Guadeloupe (n=6,189; sex ratio=0·96) and Martinique (n=10,197; sex ratio=0·88) were subjected to individual nucleic acid testing (NAT) for CHIKV RNA (n=16,386; sex ratio=0·91; pop#1; Fig 1).  Seroprevalence analyses***.*** Our objective was to follow the rise of seroprevalence at the onset of the outbreak, to estimate the final seroprevalence, and to refine the picture of epidemic kinetics and help calibrating our model by obtaining an intermediate seroprevalence value. Accordingly, a sero-epidemiological survey was conducted using a selection of samples (n=9,506) collected for NAT screening. This included donations collected in Guadeloupe and Martinique between Feb-24^th^ and July-31^st^ 2014 (n=6,812; pop#2; Fig 1), randomised samples in October 2014 (n=940; pop#3; Fig1) and January 2015 (n=1,754; pop#4; Fig 1). Seroprevalence was analysed separately for Guadeloupe and Martinique. In addition, a retrospective sero-épidemiological study was performed from donations collected in Martinique island between Aug-1^st^ 2013 and Feb-23^th^ 2014 (n=6,559; pop#5; Fig 1) in order to identify possible CHIKV circulation before the first description of autochthonous cases. | |  |
| Setting | | | 5 | Describe the setting, locations, and relevant dates, including periods of recruitment, exposure, follow-up, and data collection | | | | 8  Fig1 | | See item 4 | |  |
| Participants | | | 6 | (*a*) *Cohort study*—Give the eligibility criteria, and the sources and methods of selection of participants. Describe methods of follow-up  *Case-control study*—Give the eligibility criteria, and the sources and methods of case ascertainment and control selection. Give the rationale for the choice of cases and controls  *Cross-sectional study*—Give the eligibility criteria, and the sources and methods of selection of participants | | | | 7 | | Non-remunerated volunteers blood donors (18-70yo) recruited according to French regulatory requirements. | |  |
|  |  |  |  | (*b*) *Cohort study*—For matched studies, give matching criteria and number of exposed and unexposed  *Case-control study*—For matched studies, give matching criteria and the number of controls per case | | | | NA | | Not applicable | |  |
| Variables | | | 7 | Clearly define all outcomes, exposures, predictors, potential confounders, and effect modifiers. Give diagnostic criteria, if applicable | | | | NA | | Not applicable, Epidemiological and observational Study in Volunteer Blood Donors. | |  |
| Data sources/ measurement | | | 8* | For each variable of interest, give sources of data and details of methods of assessment (measurement). Describe comparability of assessment methods if there is more than one group | | | 7  8-9  9  9-10 | | | The information made available for analysis included date of donation, sex, age, birth place and results for ABO, Rhesus and Kell blood phenotyping.  Nucleic acids were extracted from 140µL of plasma using the NucleoSpin-96 Virus Extraction Kit (Macherey-Nagel) and a semi-automated NX-workstation (Beckman-Coulter). Real-time RT-PCR amplification was realised with the RealStar Chikungunya RT-PCR Kit 1.1 (Altona Diagnostics) and a CFX96 thermocycler (Bio-Rad).  ELISA detection of CHIKV-specific IgG was performed at the National Reference Centre for Arboviruses (French Army, Marseille, France) as previously described  Statistical analysis relied on Chi² analysis were performed online using "Chi-Square Test Calculator (http://www.socscistatistics.com/tests/). Multivariate analysis was performed using binary logistic regression with the IBM-SPSS Statistics v 23.0.0.0 software. | |  |
| Bias | | | 9 | Describe any efforts to address potential sources of bias | | | 7  10 | | | In 2014, the medical pre-donation questionnaire included a description of common CHIKV clinical symptoms. Blood donors with diagnosis or suspicion of chikungunya fever were deferred for 28 days following recovery.  Viremia duration. This estimate was therefore dependent upon the specific LOD of the detection method used. | |  |
| Study size | 10 | | | Explain how the study size was arrived at | | | 7-8  Fig1 | | | See Item 4 | |  |
| Quantitative variables | | 11 | | Explain how quantitative variables were handled in the analyses. If applicable, describe which groupings were chosen and why | | 7 | | | | Seroprevalence study. Donors recruited in the study were assigned to one of five age-groups (18-30, 31-40, 41-50, 51-60, and 61-70). | |  |
| Statistical methods | | 12 | | (*a*) Describe all statistical methods, including those used to control for confounding | | 9-10 | | | | Statistical analysis relied on Chi² analysis were performed online using "Chi-Square Test Calculator (http://www.socscistatistics.com/tests/). Multivariate analysis was performed using binary logistic regression with the IBM-SPSS Statistics v 23.0.0.0 software. | |  |
|  |  |  |  | (*b*) Describe any methods used to examine subgroups and interactions | |  | | | | See Item 12a | |  |
|  |  |  |  | (*c*) Explain how missing data were addressed | | NA | | | | Not Applicable | |  |
|  |  |  |  | (*d*) *Cohort study*—If applicable, explain how loss to follow-up was addressed  *Case-control study*—If applicable, explain how matching of cases and controls was addressed  *Cross-sectional study*—If applicable, describe analytical methods taking account of sampling strategy | | NA | | | | Not Applicable | |  |
|  |  |  |  | (*e*) Describe any sensitivity analyses | | | | 10 | | Results were considered statistically significant when p-value was lower than 0·05 | |  |
| Results | | | | | | | | | | | |  |
| Participants | | 13* | | (a) Report numbers of individuals at each stage of study—eg numbers potentially eligible, examined for eligibility, confirmed eligible, included in the study, completing follow-up, and analysed | | | | 11 | | Molecular analysis. Amongst 16,386 donations tested by RT-PCR (pop#1), 37/10,197 (0·36%) and 26/6,189 (0·42%) were positive in Martinique and Guadeloupe respectively.  Seroprevalence analysis. Monthly prevalence values of CHIKV-IgG in populations #2-5 are presented in Fig 3 for Guadeloupe and Martinique. This covers the period from Aug-1^st^ 2013 to Jan-31^st^ 2015 and includes both results from a prospective study (starting at the end of Feb-2014) and a retrospective study (Aug-2013 to Feb-2014) limited to Martinique. | |  |
|  |  |  |  | (b) Give reasons for non-participation at each stage | | | | NA | | Not Applicable | |  |
|  |  |  |  | (c) Consider use of a flow diagram | | | | Fig1 | | Figure 1 : Flow chart of sample collection | |  |
| Descriptive data | | 14* | | (a) Give characteristics of study participants (eg demographic, clinical, social) and information on exposures and potential confounders | | | | | 11  S1 Table | In the population of donors used for sero-epidemiological analyses (#pop6), the distribution of ABO blood groups was: O: 54·7%; A: 27·4%; B: 14·9%, AB: 2·90%. The prevalence values of Rhesus positive (RH+1) and Kell positive (KEL+1) phenotypes were 88·2% and 4·1%, respectively. |  |  |
|  |  |  |  | (b) Indicate number of participants with missing data for each variable of interest | | | | NA | | Not Applicable | |  |
|  |  |  |  | (c) *Cohort study*—Summarise follow-up time (eg, average and total amount) | | | | NA | | Not applicable, Epidemiological and observational Study in Volunteer Blood Donors. | |  |
| Outcome data | | 15* | | *Cohort study*—Report numbers of outcome events or summary measures over time | | | | NA | | Not applicable, Epidemiological and observational Study in Volunteer Blood Donors. | |  |
|  |  |  |  | *Case-control study—*Report numbers in each exposure category, or summary measures of exposure | | | | NA | | Not applicable, Epidemiological and observational Study in Volunteer Blood Donors. | |  |
|  |  |  |  | *Cross-sectional study—*Report numbers of outcome events or summary measures | | | | NA | | Not applicable, Epidemiological and observational Study in Volunteer Blood Donors. | |  |
| Main results | | 16 | | (*a*) Give unadjusted estimates and, if applicable, confounder-adjusted estimates and their precision (eg, 95% confidence interval). Make clear which confounders were adjusted for and why they were included | | | | Table1  11  12  14  14 | | Molecular analysis. Amongst 16,386 donations tested by RT-PCR (pop#1), 37/10,197 (0·36%) and 26/6,189 (0·42%) were positive in Martinique and Guadeloupe respectively. No significant statistical relationship between CHIKV RNA detection and age-classes or blood groups was identified.  Seroprevalence analysis. Continuous prevalence increase in both islands with a final seroprevalence of 48·1% in Guadeloupe and 41·9% in Martinique.  Significant association were identified at week level as follows (global analysis of both islands): male gender was associated with an increased risk of seropositivity; seropositivity globally increased with age.  Asymptomatic viremia duration. For a final seroprevalence of 41·9%, in the pop#1 of Martiniquan blood donors our model predicted, 15, 31, 46, and 61 RNA positive detections (with a viral load above 450 genome copies/mL of plasma) associated with asymptomatic viraemia durations of 1, 2, 3, and 4 days respectively. Based on this model, the observed number of RNA positive detections (37) corresponded to a predicted asymptomatic viraemia of *ca.* 2·5 days.  For a final seroprevalence of 48·1% in the pop#1 of Guadeloupian donors, 12, 24, 35, and 47 RNA positive detections (with a viral load above 450 genome copies/mL of plasma) were associated with asymptomatic viraemia durations of 1, 2, 3, and 4 days respectively. Thus, the observed number of RNA positive detections (26) corresponded to a predicted asymptomatic viraemia of *ca.* 2·3 days. | |  |
|  |  |  |  | (*b*) Report category boundaries when continuous variables were categorized | | | | 7  12 | | Donors recruited in the study were assigned to one of five age-groups (18-30, 31-40, 41-50, 51-60, and 61-70).  Seroprevalence analysis :Figure 4 presents the distribution of positives in age-classes in pop#4, showing *(i)* in both islands the lowest values in the 31-40yo group, *(ii)* a trend to increase in the older age-groups and *(iii)* globally higher numbers in Guadeloupe than in Martinique | |  |
|  |  |  |  | (*c*) If relevant, consider translating estimates of relative risk into absolute risk for a meaningful time period | | | | NA | | Not relevant | |  |
| Other analyses | 17 | | Report other analyses done—eg analyses of subgroups and interactions, and sensitivity analyses | | | | | | 11  12 | Molecular analysis :Monthly detection of CHIKV RNA is presented in Fig 2  Seroprevalence analysis :Figure 4 presents the distribution of positives in age-classes in pop#4, showing *(i)* in both islands the lowest values in the 31-40yo group, *(ii)* a trend to increase in the older age-groups and *(iii)* globally higher numbers in Guadeloupe than in Martinique | |  |
|  | | | Discussion | | | | | | | | | |
| Key results | 18 | | Summarise key results with reference to study objectives | | | | | | 17  17  19-20 | Important assets of the current study design were, the opportunity to compare results from similar populations in different locations, the high number of individuals enrolled, access to pre-epidemic samples, the possibility of performing individual nucleic acid tests and having access to asymptomatic or pre-symptomatic viraemiac individuals.  absence of significant circulation of the virus during several decades before the outbreak  Our study clearly shows that the risk of collecting blood from asymptomatic viraemiac patients is significant during a chikungunya fever outbreak. Modelling performed in Martiniquan and Guadeloupian blood donors suggested that the duration of asymptomatic viraemia was close to 2.5 days. | |  |
| Limitations | 19 | | Discuss limitations of the study, taking into account sources of potential bias or imprecision. Discuss both direction and magnitude of any potential bias | | | | | | 17  20 | Limitations to the interpretation of epidemiological data are therefore those of classical blood donor studies (individuals studied were 18-70 years old, and had no history of virus-like illness in the 28 days before donation).  The duration of asymptomatic viraemia was close to 2.5 days. This estimated period may be extended by the use of molecular assays with an improved limit of detection. | |  |
| Interpretation | 20 | | Give a cautious overall interpretation of results considering objectives, limitations, multiplicity of analyses, results from similar studies, and other relevant evidence | | | | | | 17  18-19  19  20  20 | The epidemic kinetics were different on the two islands, with a shorter and more intense outbreak in Guadeloupe. The peak of clinically suspected cases occurred during week-23 in both Guadeloupe (*ca.* 6,500 cases) and Martinique (*ca.* 3,250 cases). The precise origin of the observed differences is unknown. They may be related to ecological and environmental factors (e.g., climatic and geographical conditions, density and distribution of mosquito and human populations, land use...), but also to anthropogenic factors such as pressure of vector control.  The observed higher risk of CHIKV infection in males has been previously reported. However, in other studies higher prevalence in females has been reported. The reasons underlying these divergent observations remain elusive and are presumably linked with different habitats, economic factors and lifestyles. Regarding blood types and association with CHIKV infection, there is very limited information available. Kumar *et al.* claimed that Rhesus-positive individuals had increased susceptibility to acquiring CHIKV infection, but their results did not support this. In a genetic predisposition study in 100 Indian families, Lokireddy *et al.* identified infection in all Rhesus-positive blood groups but none amongst Rhesus-negative individuals. However, the proportion of Rhesus-negative individuals was too low to draw robust conclusions. The association between erythrocyte phenotypes and susceptibility to viral infection remains difficult to interpret as long as the mechanisms and cellular receptors involved in CHIKV infection are not fully identified. The risk associated with specific blood groups may be explained by several non-mutually exclusive factors: individuals with these blood groups may be more prone to mosquito bite (for biological, epidemiological or sociological reasons); alternatively, they may have different susceptibilities to infection or capacities to eliminate the virus via specific innate immune responses.  It is interesting to observe that on both islands the outbreak declined when a 40-50% herd immunity level was reached. This threshold is strikingly similar to that observed at the end of the outbreak on Reunion island (38·2%) and Mayotte (37·2%). This is despite the fact that different virus genotypes were implicated in the Caribbean and Indian Ocean epidemics. In contrast the threshold for decline was much higher in Kenya (72%), Kerala (68%) and Thailand (62·1%). This possibly reflects differing levels of social and economic development together with local efficacy of mosquito control measures.  The duration of asymptomatic viraemia was close to 2.5 days. This estimated period may be extended by the use of molecular assays with an improved limit of detection. However our results are consistent with the post-donation survey of 48 viraemiac blood donors, in which clinical symptoms were reported 1-5 days after donation (39·6% at day 1, 39·6% at day 2, 14·5% at day 3, 4·2% at day 4 and 2·1% at day 5; mean value=1·9 days). The actual mean duration of asymptomatic viraemia corresponds to this observed mean delay before the symptoms, plus the duration of viraemia prior to donation, *i.e.* it should be very close to the value provided by our model.  The length of the asymptomatic viraemic period identified in the current study is higher than previous proposals (1·5 days according to Brouard *et al.*). This divergence could reflect either different durations of asymptomatic viraemia in the case of infection by CHIKV ECSA when compared with the Asian genotype, or underestimation of the actual duration due to the limited number of cases previously analysed. | |  |
| Generalisability | 21 | | Discuss the generalisability (external validity) of the study results | | | | | | 20 | In the stressful context of emerging infectious disease outbreaks, appropriate blood donor-based studies have now been shown to be excellent first-line tools for public health surveys. This is particularly applicable to situations in which the proportion of asymptomatic individuals is high and seroprevalence information is required to estimate the attack rate as, indeed, exemplified by the currently emerging Zika virus | |  |
| Other information | | |  | |  | | | | | | | |
| Funding | 22 | | Give the source of funding and the role of the funders for the present study and, if applicable, for the original study on which the present article is based | | | | | | 26 | **Financial disclosure section on website:**  This work was supported by Etablissement Français du Sang (EFS), Alliance pour les sciences de la vie et de la santé (AVIESAN), Institut National de la Santé et de la Recherche Médicale (INSERM) and in part by the European programmes *PREDEMICS* (FP7-n°278433), *European Virus Archive goes Global* (EVAg, H2020-n°653316) and *European Network for Diagnostics of Imported Viral Diseases* (ENIVD, European Centre for Disease Prevention and Control). The work of RC was done under the frame of EurNegVec COST Action TD1303. The funders had no role in study design, data collection and analysis, decision to publish, or preparation of the manuscript. | |  |

*Give information separately for cases and controls in case-control studies and, if applicable, for exposed and unexposed groups in cohort and cross-sectional studies.

**Note:** An Explanation and Elaboration article discusses each checklist item and gives methodological background and published examples of transparent reporting. The STROBE checklist is best used in conjunction with this article (freely available on the Web sites of PLoS Medicine at http://www.plosmedicine.org/, Annals of Internal Medicine at http://www.annals.org/, and Epidemiology at http://www.epidem.com/). Information on the STROBE Initiative is available at www.strobe-statement.org.
